# The Role of Dynamic Susceptibility Contrast-Enhanced Perfusion MR Imaging in Differentiating between Infectious and Neoplastic Focal Brain Lesions: Results from a Cohort of 100 Consecutive Patients

**DOI:** 10.1371/journal.pone.0081509

**Published:** 2013-12-06

**Authors:** Valdeci Hélio Floriano, Ulysses S. Torres, Antonio Ronaldo Spotti, José Roberto Lopes Ferraz-Filho, Waldir Antônio Tognola

**Affiliations:** 1 Department of Radiology, Hospital de Base, São José do Rio Preto Medical School (FAMERP), São José do Rio Preto, São Paulo, Brazil; 2 Department of Neurological Sciences, Hospital de Base, São José do Rio Preto Medical School (FAMERP), São José do Rio Preto, São Paulo, Brazil; University of Minnesota, United States of America

## Abstract

**Background and Purpose:**

Differentiating between infectious and neoplastic focal brain lesions that are detected by conventional structural magnetic resonance imaging (MRI) may be a challenge in routine practice. Brain perfusion-weighted MRI (PWI) may be employed as a complementary non-invasive tool, providing relevant data on hemodynamic parameters, such as the degree of angiogenesis of lesions. We aimed to employ dynamic susceptibility contrast-enhanced perfusion MR imaging (DSC-MRI) to differentiate between infectious and neoplastic brain lesions by investigating brain microcirculation changes.

**Materials and Methods:**

DSC-MRI perfusion studies of one hundred consecutive patients with non-cortical neoplastic (n = 54) and infectious (n = 46) lesions were retrospectively assessed. MRI examinations were performed using a 1.5-T scanner. A preload of paramagnetic contrast agent (gadolinium) was administered 30 seconds before acquisition of dynamic images, followed by a standard dose 10 seconds after starting imaging acquisitions. The relative cerebral blood volume (rCBV) values were determined by calculating the regional cerebral blood volume in the solid areas of lesions, normalized to that of the contralateral normal-appearing white matter. Discriminant analyses were performed to determine the cutoff point of rCBV values that would allow the differentiation of neoplastic from infectious lesions and to assess the corresponding diagnostic performance of rCBV when using this cutoff value.

**Results:**

Neoplastic lesions had higher rCBV values (4.28±2.11) than infectious lesions (0.63±0.49) (*p*<0.001). When using an rCBV value <1.3 as the parameter to define infectious lesions, the sensitivity of the method was 97.8% and the specificity was 92.6%, with a positive predictive value of 91.8%, a negative predictive value of 98.0%, and an accuracy of 95.0%.

**Conclusion:**

PWI is a useful complementary tool in distinguishing between infectious and neoplastic brain lesions; an elevated discriminatory value for diagnosis of infectious brain lesions was observed in this sample of patients when the rCBV cutoff value was set to 1.3.

## Introduction

Differentiating between infectious and neoplastic focal brain lesions that are detected by imaging examinations is an important role usually expected from radiologists. Differentiation has critical implications for the appropriate workup and management of patients, but it also frequently poses a significant challenge. Although conventional techniques of structural MRI with gadolinium-based contrast agents have a fundamental and well-established role in the diagnosis, evaluation and follow-up of brain lesions as a consequence of their excellent delineation of anatomical and morphological data, tissue contrast and multiplanar acquisitions capability [1]–[3], these same techniques have some inherent limitations, including difficulties in assessing the grade, type, extension and limits of tumoral lesions, as well as a lack of data on physiological parameters such as tumoral vascularity and metabolism [1], [2], [4]–[6]. Due to these limitations, these conventional techniques have a limited role in reliably differentiating tumors from tumor-like conditions [5], [6].

Specific types of central nervous system (CNS) infections constitute a significant spectrum of these tumor like-conditions. CNS infections represent an important health problem, mainly in developing countries, and their incidence has been rising worldwide during the last few decades as a result of the acquired immunodeficiency syndrome (AIDS) epidemic, the use of immunosuppressive drugs and the emergence of multidrug-resistant strains [7], [8]. Because most causes of CNS infections are potentially treatable, an early and correct diagnosis is essential, and neuroimaging plays a pivotal role in this context [7]. Nonetheless, imaging findings depicted by conventional MRI sequences are often insufficient to clearly discriminate infectious lesions from neoplastic processes [5], [9]–[12], and some forms of CNS infections eventually may be incorrectly diagnosed as neoplasms [5]. Conditions such as pyogenic abscesses, toxoplasmosis, tuberculosis, cysticercosis, fungal infections and syphilis can mimic brain neoplasms on neuroimaging, while some brain neoplasms (low-grade gliomas, glioblastomas, lymphomas and metastases) may be not associated with typical tumefactive lesions [13].

In an attempt to avoid unnecessary biopsies (an invasive procedure associated with morbidity risk and transient neurological deficits [14]) in patients with benign lesions, advanced MRI techniques such as PWI, diffusion-weighted imaging (DWI) and proton MR spectroscopy may provide additional information aimed at improving the characterization of these lesions, thus narrowing the differential diagnoses [1]–[7], [9]–[13].

Currently, there are two main approaches clinically employed to perform PW-MRI, T1-weighted (dynamic contrast-enhanced MRI, DCE-MRI) or T2-weighted (DSC-MRI). DCE-MRI relies on the acquisition of serial T1-WI (weighted-images) before, during and after the injection of contrast medium, aiming at characterizing tumor microcirculation (e.g., permeability) [15]. By applying a contrast kinetics analysis to the concentration-time curves measured in each pixel in a given tissue, this technique yields to calculate parameters related to tissue permeability and blood flow (denoted by the transfer constant, *k^trans^*), as well as to the fractional volume of extravascular-extracellular space (*v_e_*), for example [15]. The T2-W approach of DSC-MRI, otherwise, is a widely accepted technique for PWI that allows characterization of brain hemodynamics in normal and pathological conditions (including intracranial masses), making it an important tool for assessment of brain tumors [16]. This approach is based on the principle that the intense cellular growth and increased cellular turnover observed in neoplastic lesions leads to increased metabolic demands and a state of hypoxia and hypoglycemia within the tumor. The hypoxia and hypoglycemia then stimulate the production of angiogenic cytokines (e.g., vascular endothelial growth factor, VEGF) and a consequent angiogenesis [1]. The resultant higher capillary density within glial tumors as revealed by PW-MRI correlates well with histological and angiographic vascularities [17]. This technique allows, therefore, the derivation of relative cerebral blood volume (rCBV) maps based on the susceptibility effect determined by the passage of a paramagnetic MRI contrast agent through brain tissue [12], [18]. DSC-MRI has two variants, the T2*-W method (gradient-echo sequences) and the T2-W method (spin-echo sequences) [19]. Although both T2*- and T2-weighting provide reliable CBV measurements [1], [19], spin-echo sequences are mainly sensitive to smaller vessels (<20 µm) and are less susceptible to artifacts, whereas gradient-echo sequences are less dependent on the vessel size [1], [19], [20].

It should be stressed, however, that increased tumor vascularity is not necessarily synonymous with malignancy [21]. Indeed, benign hypervascularized tumors such as meningiomas and pituitary adenomas, for example, also show high rCBV values [22]. Notwithstanding, rCBV values correlates well with malignancy in gliomas, being widely employed for grading, differentiating and biopsy planning in such cases, as well as to determine prognosis and treatment monitoring [19], [22]; an exception to this are some low-grade oligodendrogliomas, which can demonstrate high rCBV values despite of their histological grading [1].

In lesions with a severe blood-brain barrier (BBB) breakdown, paramagnetic contrast agent extravasates from the vascular compartment into the interstitium, which promotes an unwanted T1 shortening effect in addition to the T2 or T2* effects; this T1 effect raises the signal intensity above baseline and is interpreted by the algorithm for quantification of rCBV as a negative blood volume, thus leading to an underestimation of rCBV [1], [4], [19]. One approach to address this problem is to administer a preload of contrast agent (a small dose of gadolinium) to presaturate the interstitium and to elevate the baseline, thus diminishing the T1-leakage effects [1], [19], [23].

Despite being widely employed in tumor assessment, only a few published studies have formally and directly evaluated the role of the DSC-MRI technique in differentiating between neoplastic and infectious brain lesions [10]–[12], [24], [25]. Some of these studies employed relatively small samples or included a reduced number of patients with infectious lesions, and each reached distinct results. We hypothesized that DSC-MRI can aid in distinguishing between infectious and neoplastic brain lesions and that rCBV values found in infectious lesions would be decreased compared with neoplastic lesions. In this study, we sought to investigate this question by employing one of the largest series in the literature.

## Materials and Methods

### Ethics Statement

The study protocol was approved by the Institutional Ethics Committee of the São José do Rio Preto Medical School, Brazil. Written informed consent was obtained from all subjects before imaging acquisition.

### Patients

We retrospectively analyzed a total of 100 consecutive patients (mean age, 47.7±14.8 years; age range, 18–84 years; 69 men) with new and untreated neoplastic or infectious brain lesions who underwent MRI examinations at our tertiary center in the setting of a clinical suspicion of a mass lesion. The patients were divided into two groups. Group 1 (54 patients, mean age 53.4±15.3 years; age range, 18–80 years; 37 men) comprised those with neoplastic lesions, either primary (38 patients) or metastatic (16 patients). All primary brain tumors were diagnosed histopathologically and graded according to the World Health Organization (WHO) classification scheme [26], as follows: grade II gliomas (n = 6; 3 astrocytomas and 3 oligodendrogliomas), grade III gliomas (n = 9; 7 anaplastic astrocytomas, 1 oligodendroglioma and 1 anaplastic ependymoma) and grade IV gliomas (glioblastoma multiforme) (n = 23). All metastatic lesions were biopsied; these lesions originated from lung (n = 10), breast (n = 1), gastric (n = 1) and rectal (n = 1) cancers, melanoma (n = 1) and non-Hodgkin’s lymphoma (n = 1); one metastatic lesion was of unknown primary origin. Group 2 (46 patients, mean age 40.5±11.7 years; age range, 18–84 years; 32 men) comprised patients with infectious lesions, distributed as follows: toxoplasmosis (n = 38), pyogenic abscess (n = 2), cryptococcomas (n = 2), cysticercosis (n = 2), tuberculoma (n = 1) and paracoccidioidomycosis (n = 1). Diagnoses of infectious lesions were reached on the basis of clinical history, clinical presentation, cerebrospinal fluid analysis, imaging features and clinical-neuroradiological improvement at follow-up after specific treatment. Overall, 59% of patients had single lesions (46 patients in the neoplastic group and 13 patients in the infectious group), and 41% had two or more lesions (8 patients in the neoplastic group and 33 patients in the infectious group).

### MRI Acquisition

We performed the brain examinations on a 1.5-T scanner (Gyroscan Intera, Philips Medical Systems, Best, the Netherlands) using a standard quadrature birdcage head coil. The routine MRI protocol consisted of axial fluid-attenuated inversion recovery (FLAIR) images (TR/TE/TI = 6000/120/2000 ms; slice thickness, 5 mm; slice interval, 1 mm; field of view (FOV), 250×80 mm; matrix, 256×512; number of signal averages (NSA) = 3), axial turbo spin-echo (TSE) T2-W (TR/TE = 5114/110 ms; slice thickness, 5 mm; slice interval, 1 mm; FOV, 250×70 mm; matrix, 400×512; NSA = 2), axial single-shot spin-echo echo-planar DW imaging with diffusion sensitivities of b = 0 mm^2^/s and b = 1,000 mm^2^/s (TR/TE = 2926/75 ms; slice thickness, 5 mm; slice interval, 1 mm; FOV, 230×100 mm; matrix, 128×512; NSA = 3), axial T2*-W (TR/TE = 1740/30 ms; slice thickness, 5 mm; slice interval, 0.5 mm; FOV, 250×100 mm; matrix, 256×512; NSA = 1), sagittal T1-W before and after administration of paramagnetic contrast agent (TR/TE = 529/15 ms; slice thickness, 5 mm; slice interval, 1 mm; FOV, 250×85 mm; matrix, 256×512; NSA = 2), coronal T1-W after administration of paramagnetic contrast agent (TR/TE = 522/15 ms; slice thickness, 5 mm; slice interval, 1 mm; FOV, 190×90 mm; matrix, 224×512; NSA = 2) and axial T1-W images with use of magnetization transfer after administration of paramagnetic contrast agent (TR/TE = 435/15 ms; slice thickness, 5 mm; slice interval, 1 mm; FOV, 250×70 mm; matrix, 256×512; NSA = 2).

DSC-MRI examinations were performed with spin-echo echo-planar T2-W sequence using the following parameters: TR/TE = 956/42 ms, slice thickness = 7 mm; slice interval = 0 mm; FOV, 230×230 mm; flip angle, 40°; matrix, 128×128. A series of images (10 slices, 100 images/slice) was obtained within 100 seconds. Intravenous administration of paramagnetic contrast agent (gadoterate meglumine, Dotarem®, Guerbet) was performed in the antecubital vein with an automatic injector at a flow velocity of 5 ml/s through an 18- to 20-gauge catheter in two separate doses: a smaller initial dose (or preload) (0.05 mmol/kg) 30 seconds before acquisition of dynamic images to presaturate the interstitium and minimize T1-shortening effects and a second (standard) dose (0.2 mmol/kg) 10 seconds after starting imaging acquisitions, followed by a bolus injection of 20 ml of saline flush.

### Image Processing

All perfusion data were transferred to a workstation (EasyVision, Philips Medical Systems, Best, the Netherlands) and processed with a commercially available dedicated software package (ViewForum R5.1, Philips Medical Systems, Best, the Netherlands). Briefly, the signal intensity time course was obtained from each pixel in the echo-planar image. After, time-dependent concentration of the paramagnetic contrast agent in the tissue was modeled as a function of the injected bolus, cerebral blood flow, and the residual contrast in the tissue at a given time, being inferred from the effects of contrast medium on the relaxation rate (ΔR2). These changes in ΔR2 are assumed to be linearly proportional to the concentration of the contrast medium in the tissue [12], [27]. The following equation was used: ΔR2 = −ln(S/S_0_)/TE, where *ln* is the natural logarithm, *S* is signal intensity and *S_0_* is baseline signal intensity. Subsequently, by considering the limits of integration between the end of the baseline and the through signal intensity (area under the peak), the rCBV maps were generated [12], [27]. As previously addressed, because the BBB may be disrupted in neoplastic and infectious brain lesions, the assumptions of a negligible T1 effect and that the contrast medium remains intravascular are incorrect, thus the kinetic model aforementioned is not valid (T1 shortening will mask the signal loss due to T2 and rCBV will be underestimated) [27]. Although the contrast-leakage correction may be performed using mathematic models [28], we used a common approach of minimizing this effect by administering a preload of contrast medium. Hence, this preload dose can sufficiently produce a T1 shortening, such that this effect may be negligible when the main bolus is used to quantify perfusion [27]. In this sense, for practical purposes, the preload was used solely to neglect the extra signal enhancement, and only the passage of main bolus was considered in the calculations to quantify perfusion.

The maximum rCBV was calculated in the color-coded maps of cerebral blood volume by placing standardized regions of interest (ROI) of 50 mm^2^ in the peripheral solid portions of the lesions and in the normal-appearing contralateral white matter in the same slice as the lesion, according to the following equation*: rCBV = regional CBV_lesion_*/*regional CBV_contralateral white matter_*.

### Statistical Analysis

We compared the rCBV values between the two groups (neoplastic *versus* infectious) using Student’s t-test. The rCBV values are presented on the basis of the averages and standard deviations. We also calculated the cutoff point of rCBV values for PWI that would allow differentiation of neoplastic and infectious lesions. We employed receiver operating characteristic (ROC) curve analysis to assess the performance of a simple diagnostic test designed to correctly classify a lesion as infectious rather than neoplastic using a given rCBV value in relation to the defined cutoff. We then calculated sensitivity, specificity, positive predictive value, negative predictive value and accuracy (as well as the corresponding estimated confidence intervals) of this diagnostic test when employing the retrieved cutoff rCBV value (all values are presented as %). A *p-*value <0.05 was considered statistically significant. Statistical analysis was performed using the Minitab 15® software (Minitab Inc., Minneapolis, USA).

## Results

**Figure 1:**
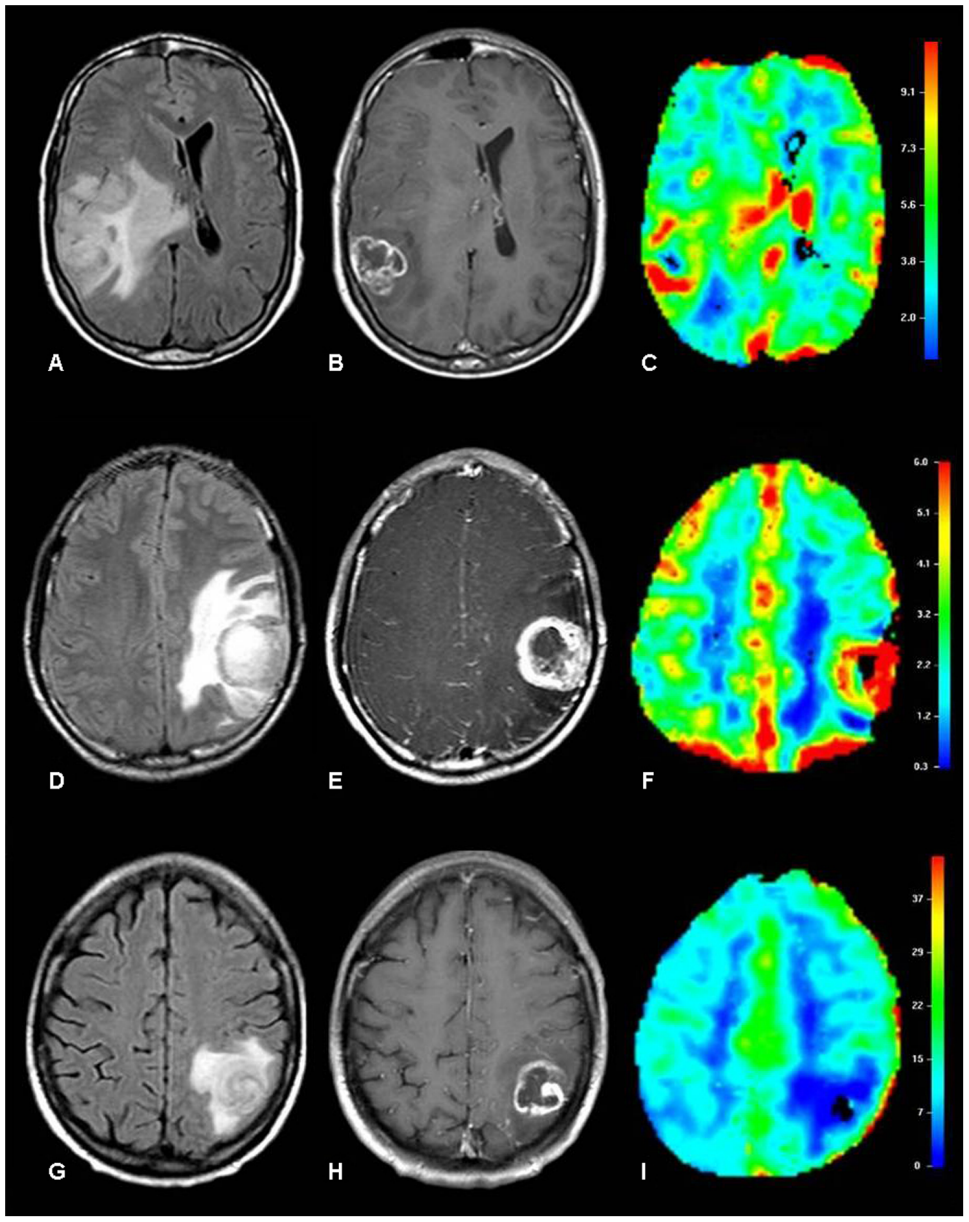
Examples of rCBV maps of neoplastic and infectious lesions. Glioblastoma multiforme in a 49-year-old man (A,B,C). Axial FLAIR image shows a mass lesion in the right parietal lobe surrounded by infiltrating and vasogenic edema (A), with irregular peripheral enhancement on axial contrast-enhanced T1-W image (B) and increased vascularity on the color-coded rCBV map (C). Metastatic melanoma in an 18-year-old man (D,E,F). Axial FLAIR image depicts a mass lesion in the left parietal lobe with perilesional vasogenic edema (D), irregular peripheral rim enhancement on the axial post-contrast T1-W image (E) and increased vascularity on the color-coded rCBV map (F). Cerebral toxoplasmosis in a 54-year-old woman (G,H,I). Axial FLAIR image shows a mass lesion in the left parietal lobe with surrounding vasogenic edema (G), irregular peripheral rim enhancement on the axial post-contrast T1-W image (H) and hypovascularity on the color-coded rCBV map (I).

Neoplastic lesions had higher rCBV values than infectious lesions (*p*<0.001). No significant difference was found regarding rCBV values when comparing primary and metastatic lesions within the neoplastic group (*p* = 0.676). Univariate analysis of rCBV values for each group of lesions and specifically for the two subsets within the neoplastic group is summarized in Table 1 (ranges, means and standard deviations). Examples of rCBV maps of neoplastic (primary and metastatic) and infectious lesions are shown in Figure 1.

**Table 1:** Univariate analysis of rCBV values for each group of lesions (neoplastic and infectious) and for the two subsets within the neoplastic group (primary and metastatic lesions).

Group	*n*	Mean ± SD	Range	*p* value
Infectious	46	0.63±0.49	0.3–3.5	<0.001
Neoplastic	54	4.28±2.11	0.4–9.7	
Primary neoplastic	38	4.37±2.10	0.4–9.7	0.676
Metastatic	16	4.07±2.20	1.1–8.7	

SD: standard deviation.

**Figure 2:**
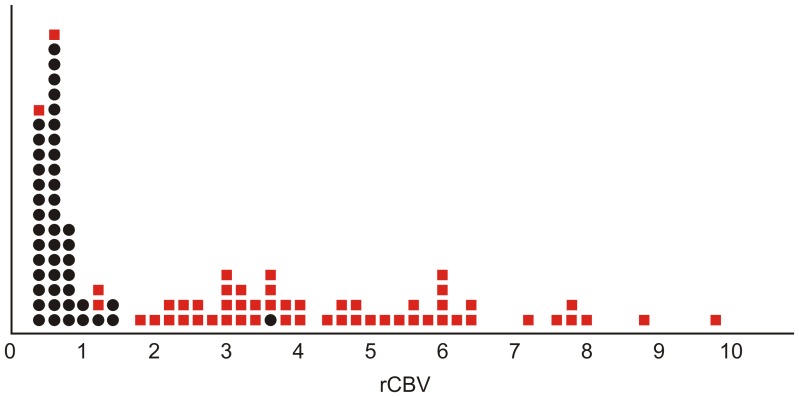
rCBV values (*x*-axis) obtained for each patient are represented as individual points and classified according to the characteristics of the lesions: neoplastic (red squares) or infectious (black dots). Note that the points corresponding to infectious lesions are mainly concentrated in the lower values of rCBV, whereas the points for the group of neoplastic lesions are more dispersed and show a trend of higher rCBV values.

Individual rCBV values obtained for each patient and classified by groups (neoplastic or infectious) are graphically displayed in Figure 2. It is notable that the graphical points for the group of infectious lesions are mainly concentrated in the lower values of rCBV, whereas the graphical points for the group of neoplastic lesions are more dispersed and tend to have higher values of rCBV. The mean rCBV values according to each type of lesion are detailed in Table 2.

**Table 2:** Mean rCBV values according to type of lesion.

	Lesion	Number of patients	rCBV value (mean ± SD)*
**Neoplastic**	Grade II gliomas	6	2.78±1.92
	Grade III gliomas	9	3.95±2.36
	Grade IV gliomas	23	4.95±1.85
	Metastases	16	4.07±2.20
**Infectious**	Toxoplasmosis	38	0.63±0.49
	Pyogenic abscesses	2	0.75±0.63
	Cryptococcomas	2	0.50±0.14
	Cysticercosis	2	0.50±0.14
	Tuberculoma	1	3.50
	Paracoccidioidomycosis	1	0.70

* Results are presented as mean ± SD, except for the cases of tuberculoma and paracoccidioidomycosis (one patient in each category), which are presented as single values.

**Figure 3:**
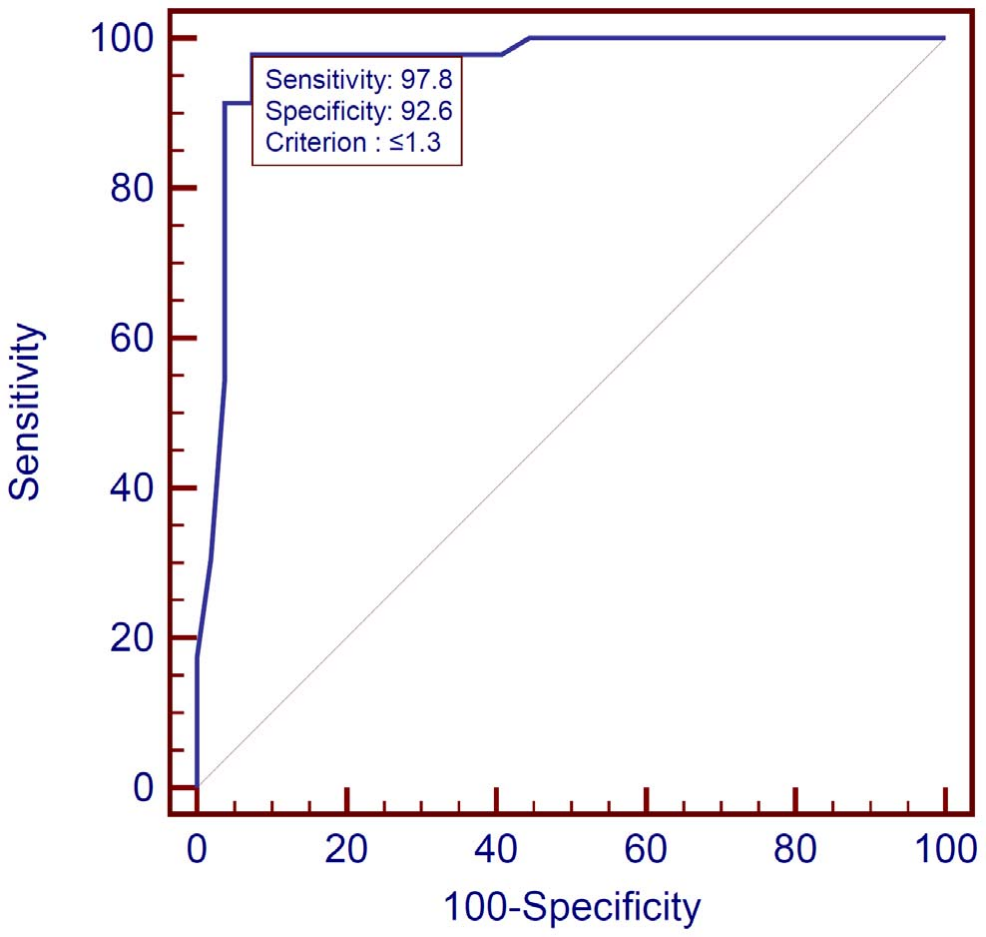
ROC curve representing the discriminatory capability of rCBV in correctly classifying a lesion as infectious using a cutoff point of 1.3. The large area under the curve (0.964) indicates the good discriminatory ability of the method.

**Figure 4:**
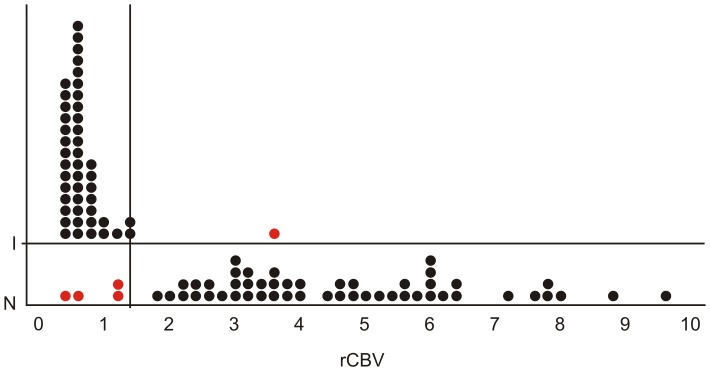
A cutoff point for rCBV values (*x-*axis) of 1.3 allows the correct classification (black dots) of all but one infectious lesions and almost all neoplastic lesions; four cases of neoplastic lesions were misclassified as being infectious (red dots). *I = infectious; N = neoplastic*.

Evaluation of discriminatory capability of the DSC-MRI method through ROC analysis defined the rCBV value that better allowed a correct differentiation of infectious from neoplastic lesions, retrieving a threshold of 1.3. The ROC curve representing the discriminatory capability of an rCBV value of <1.3 to correctly classify a lesion as infectious is shown in Figure 3. Table 3 demonstrates the corresponding sensitivity, specificity, positive predictive value, negative predictive value, accuracy and confidence intervals of this diagnostic test. The discriminant function analysis misclassified four cases of neoplastic lesions as being infectious because their rCBV values were <1.3, and one case of infectious lesion as being neoplastic because its value was >1.3, as depicted by Figure 4.

**Table 3:** Diagnostic performance of rCBV for the diagnosis of infectious lesions with a cutoff point of 1.3.

Measure	Value (%)	95% CI
Sensitivity	97.8	(88.5; 99.9)
Specificity	92.6	(82.1; 97.9)
PPV	91.8	(80.4; 97.7)
NPV	98.0	(89.5; 99.9)
Accuracy	95.0	(90.7; 99.3)

PPV: positive predictive value; NPV: negative predictive value; CI: confidence interval.

## Discussion

Our results demonstrated statistically significant lower rCBV values in brain infectious lesions than in neoplastic lesions, and a good ability of PWI to successfully distinguish between these two conditions, with elevated sensitivity, specificity, positive and negative predictive, and accuracy values. Haris *et al.* [11], in a study comprising 103 patients (77 with neoplastic lesions and 26 patients with infectious lesions), found mean rCBV values of 3.66±0.58 in patients with infectious lesions, which were lower than the values observed in patients with high-grade gliomas (5.78±1.11) but higher than the values found in patients with low-grade gliomas (2.52±0.74); the differences among all three groups were statistically significant.

A study by Hakyemez *et al*. [24], which involved a total of 105 patients with varied brain mass lesions (only four cases of infectious lesions, i.e., pyogenic abscesses), found mean rCBV values of 5.76±3.35 in high-grade gliomas (n = 26), 5.27±3.22 in metastatic lesions (n = 25), 1.69±0.51 in low-grade gliomas (n = 11) and 0.76±0.12 in pyogenic abscesses; high-grade gliomas and metastases could be successfully differentiated from abscesses on the basis of PWI (*p*<0.0001). Hourani *et al.* [5], although not specifically studying infectious lesions, compared patients with neoplastic brain lesions (n = 36) and nonneoplastic brain lesions (n = 33, including stroke, demyelinating disease, multiple sclerosis and acute disseminated encephalomyelitis, among others); these authors found higher mean rCBV values in neoplastic lesions (4.11±3.14) in comparison to nonneoplastic lesions (1.00±0.39); however, low-grade tumors (1.5±1.2) could not be differentiated from nonneoplastic lesions based on PWI (*p* = 0.73). We were not able to specifically compare between low-grade gliomas and high-grade gliomas or infectious lesions because of the limited number of patients with low-grade tumors in our sample (6 patients).

In the study by Hourani *et al*. [5], a threshold rCBV value of 1.5 was suggested for differentiating between neoplastic and nonneoplastic lesions, with a sensitivity, specificity, positive predictive value and negative predictive value of 77.8%, 91.7%, 93.3% and 91.7%, respectively. This suggested cutoff value, which is slightly higher than that found in our own study, and the distinct mean rCBV values found in several different studies may be more useful as qualitative indicators of trends, and should not be taken into an absolute or definitive numeric perspective. The numeric variations may have been affected by multiple factors, including those inherent to the sample (size, heterogeneity and variability of included lesions), subjects (age, immunological status), lesions (type, biological behavior, morphology, dimensions, degree of angiogenesis), scanning protocols, perfusion technique (T1, gradient-echo or spin-echo sequences, arterial spin labeling), administration or not of the preload of paramagnetic contrast agent, and imaging processing (employed software), among others.

Several studies have demonstrated a direct correlation between mean rCBV values and histological grading of gliomas, with high-grade gliomas showing higher rCBV values than low-grade gliomas and nonneoplastic lesions [1]–[3], [5], [17], [19]–[21], [29]. Differentiating between high-grade gliomas and solitary metastasis may require analysis of peritumoral areas. Hyperintense peritumoral regions on T2-W images may represent vasogenic edema secondary to increased capillary endothelium permeability in metastatic tumors but could also be the result of tumoral infiltration in primary gliomas [30]. In this sense, significantly higher rCBV values have been reported [30] in the perilesional areas of high-grade gliomas (possibly explained by this infiltrative feature) compared with metastases (possibly because of a lack of tumoral infiltration as well as local compression of the microcirculation by extravasated edema fluid) [30]. The mean rCBV values of metastatic lesions in our study were based only on analyses of solid peripheral areas without inclusion of perilesional regions; thus, we did not find statistically significant differences between these two conditions.

Toxoplasmosis was the most frequent CNS infection in our sample of patients. With effect, toxoplasmosis is the most common brain mass lesion in patients with AIDS [8]. MRI is the most sensitive imaging approach for this diagnosis, demonstrating multiple, nodular or ring-like enhancing lesions with surrounding vasogenic edema, located both in the white matter and deep gray matter [9]. Nonetheless, MRI findings are not pathognomonic for toxoplasmosis, as similar findings may be observed in various infectious or noninfectious brain diseases, such as tuberculomas, cryptococcomas, lymphoma and metastases [9]. A presumptive diagnosis of toxoplasmosis is made on the basis of the clinical setting, the imaging findings and a positive serologic test; clinical and neuroradiological improvement at follow-up after empiric antitoxoplasma treatment is also a reliable indicator of toxoplasmosis [9]. On PW-MRI, toxoplasmosis usually shows low rCBV values within the lesion and in the surrounding edema, most likely because of a lack of vascularization within the lesion as well as vasoconstriction in the surrounding edema due to increased interstitial pressure [31]. In the HIV-infected patient, the major differential diagnosis is primary CNS lymphoma [8], [9].

Interestingly, CNS lymphomas are an example of how rCBV values may be different depending on whether DSC perfusion is performed with the administration of a preload of contrast agent or not. When a preload dose is administered, CNS lymphomas may present focal areas of increased rCBV values; however, this is not due to the typical hypervascularity of an active neoplastic process (as neovascularization is not a prominent histologic feature in these tumors), but due to a greater degree of BBB destruction and higher vascular permeability [24], [28], [32]. This feature could be useful in the differentiation of lymphomas from neurotoxoplasmosis, for example, which usually show low rCBV values. On the other hand, when the leakage correction is not performed (by injecting a preload dose, for example), the rCBV in CNS lymphomas may be underestimated, resulting in lower values [28], [32]. Hence, this type of PWI may be helpful in the differential diagnosis of CNS lymphomas from high grade gliomas, metastases, or meningiomas.

Tuberculomas are a type of infectious brain lesion with interesting PWI findings. As for other infectious brain mass lesions, differentiation of tuberculomas from primary tumors is often difficult on conventional structural MRI, and multiple tuberculomas may mimic metastatic lesions [33]. Contrary to what is expected for an infectious lesion, however, Batra and Tripathi [33] found higher rCBV values, similar to those reported for gliomas, in the peripheral solid areas of tuberculomas and low rCBV values in the nonenhanced central areas of the lesions and in the surrounding edema. These findings are consistent with the histological features of tubercular granulomas, which are characterized by central caseation necrosis and inflammatory hypervascularity with subsequent reactive wall neovascularization [33]. Again, decreased rCBV values in the perilesional edema may be explained by the vasoconstriction induced by increased interstitial pressure in the setting of edema [33]. Indeed, the only case of tuberculoma in our sample showed an elevated rCBV value (3.5), in accordance with the results of Batra and Tripathi [33], which made this the only patient with an infectious lesion being misclassified within the neoplastic group.

Haris *et al.* [11] found a higher mean rCBV value in their group of patients with infectious lesions (3.66±0.58) than that observed within our infectious group (0.63±0.49), a difference that can be partially explained by the elevated number of tuberculomas in the infectious group of their study (69%) and the elevated number of toxoplasmosis cases in our infectious group (82%). Additionally, other factors such as patient immune status, features of each specific pathogen and the evolutive stage of the infectious process may contribute to the variability of mean rCBV values in infectious lesions [9], [21].

In conclusion, our study provides additional data on the usefulness of DSC-MRI in correctly differentiating between infectious and neoplastic brain lesions and as a complementary tool to routine structural MRI. An exception in our study was low-grade gliomas, which, in our sample of patients, had rCBV values overlapping with those found in infectious lesions. Recently, with the more widespread availability of high-field MRI scanners at 3-T in the clinical practice, allowing valuable effects such as higher signal-to-noise ratio, better spatial resolution of images, shorter scan time and the concurrent acquisition of conventional structural imaging and advanced techniques such as PWI, DWI and spectroscopy [34], it is expected an increasingly facilitated multiparametric approach to brain mass lesions, which may improve diagnostic accuracy. Furthermore, arterial spin labeling (ASL), an attractive non-invasive technique that measures blood flow by using arterial blood water as an endogenous contrast agent (thus not requiring the administration of exogenous gadolinium-based contrast agent, which carries the risk of nephrogenic systemic fibrosis in patients with poor renal function), previously had limited clinical use in routine practice, but as 3-T MRI scanners have become more widely available (addressing 1.5-T limitations, such as low SNR) [34], this technique may now be practical and offer an important impact in the assessment of neoplastic and infectious brain lesions.
